# Hand hygiene practice and associated factors among rural communities in northwest Ethiopia

**DOI:** 10.1038/s41598-023-30925-0

**Published:** 2023-03-15

**Authors:** Zemichael Gizaw, Negesu Gizaw Demissie, Mulat Gebrehiwot, Bikes Destaw, Adane Nigusie

**Affiliations:** 1 Department of Environmental and Occupational Health and Safety, Institute of Public Health, College of Medicine and Health Sciences, University of Gondar, Gondar, Ethiopia; 2 Department of Medical Nursing, School of Nursing, College of Medicine and Health Sciences, University of Gondar, Gondar, Ethiopia; 3 Department of Health Education and Behavioral Sciences, Institute of Public Health, College of Medicine and Health Sciences, University of Gondar, Gondar, Ethiopia

**Keywords:** Medical research, Risk factors

## Abstract

This community-based cross-sectional study was conducted among 1190 randomly selected rural households in northwest Ethiopia to assess hand hygiene practice and associated factors. Frequent handwashing with rubbing agents, drying mechanisms; and condition of fingernails were used to assess hand hygiene practice. Multivariable binary logistic regression analysis was used to identify factors associated with hand hygiene and statistically significant association was declared on the basis of adjusted odds ratio (AOR) with 95% confidence interval (CI) and p-values < 0.05. Results showed that 28.8% (95% CI 26.2, 31.4%) of the households had good hand hygiene practice. Good hand hygiene practice was significantly associated with formal education attended household heads (AOR 1.79, 95% CI 1.33, 2.40), family discussion on sanitation (AOR 1.56, 95% CI 1.08, 2.26), provision of health education (AOR 2.23, 95% CI 1.62, 3.06), and availability of water (AOR 3.51, 95% CI 1.02, 12.05). In conclusion, about one-third of the rural households had good hand hygiene practice and more than two-third had poor hand hygiene practice in the study area, and this may imply that hands in the area may play roles in spreading infections in the community. Therefore, people need to be informed to always keep their hand hygiene good.

## Introduction

Hand hygiene which is a method of hand cleaning that significantly reduces microorganisms from the hands, is one of the most important preventative measures for infectious disease transmission. Hand hygiene procedures include the use of water and plain or antimicrobial soaps, and alcohol-based hand rubs for the purpose of removing or destroying transient microorganisms and reduce resident flora^1–3^. In the absence of alcohol-based hand rubs and soap, the use of readily accessible rubbing agents such as sand, ash, soil, and leaves is an alternative method to perform hand hygiene^4^. Improved hand hygiene can reduce the risk of acquiring gastrointestinal and respiratory tract infections up to 50–60%^5^.

**Figure 1:**
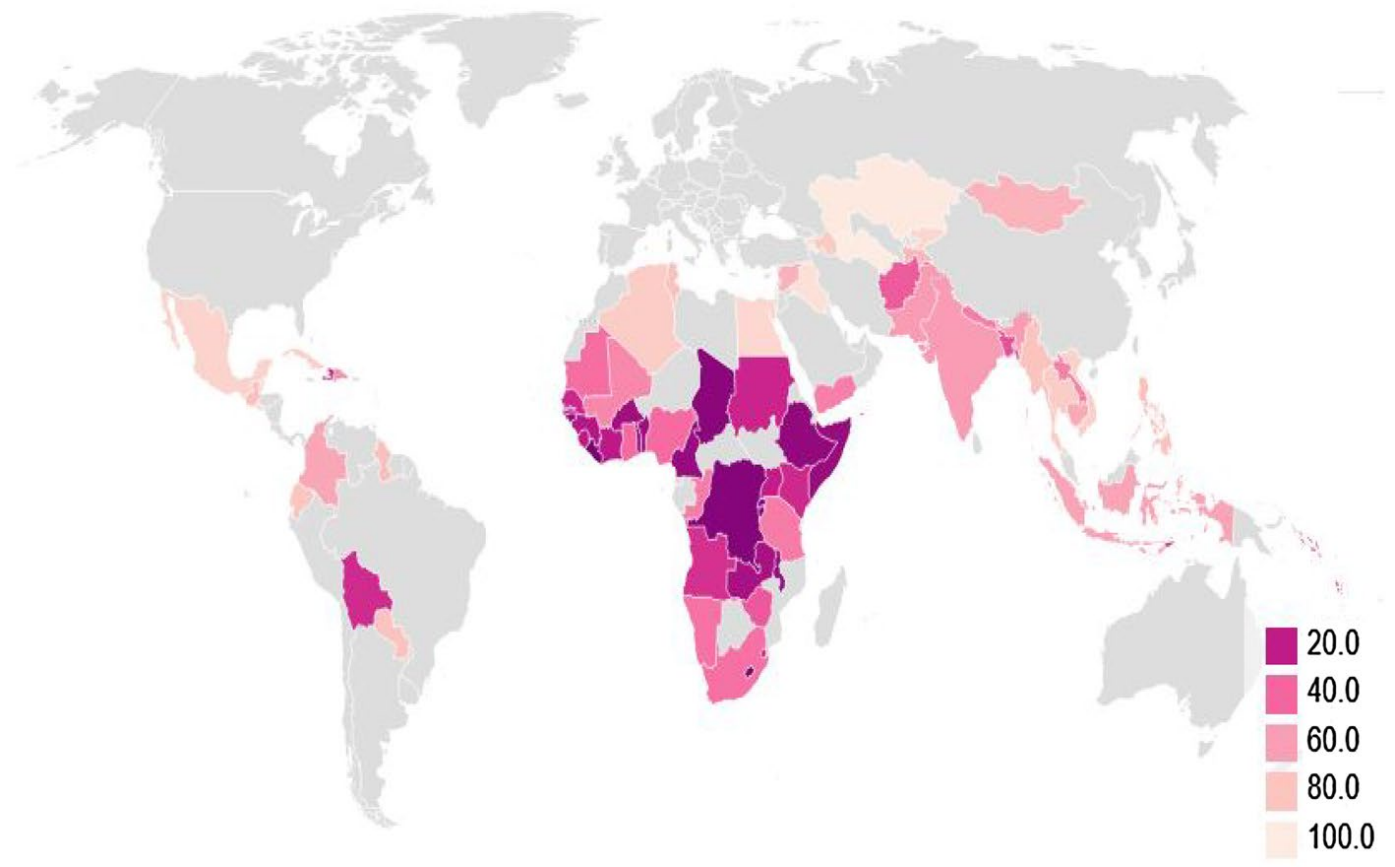
People with basic handwashing facilities including soap and water, 2017 (% of population) (Source: JMP (WHO/UNICEF); WDI (SH.STA.HYGN.ZS))^9^.

Hand hygiene refers to removing or killing microorganisms on the hands. When performed correctly, hand hygiene is the single most effective way to prevent the spread of communicable diseases and infections. Hand hygiene may be performed either by washing using soap and running water or using alcohol-based hand rubs. The mechanical action of washing, rinsing, and drying removes transient bacteria present on the hands. Hand washing with soap and running water must be performed whenever hands are visibly soiled^6,7^. However, globally, around 3 in 10 people or 2.3 billion people do not have handwashing facilities with water and soap available at home. However, absence of handwashing facilities with water and soup is disproportionately high in the least developed countries, with over 6 in 10 people without access to basic hand hygiene^8^. Countries with low access to basic handwashing facilities are concentrated in Sub-Saharan Africa as illustrated in Fig. 1. Moreover, those living in rural areas are generally more disadvantaged when it comes to accessing basic handwashing facilities than people living in urban areas^9^.

Hand hygiene is a simple and effective public health measure that everybody should practice to prevent the spread of infections. Rural communities in the study area, however, have poor perception about the importance of hand hygiene and have poor awareness on effective hand hygiene procedures. For instance, a randomized controlled trial study conducted in a rural setting of northwest Ethiopia reported that 60 (27.3%) and 53 (24.1%) of women in the intervention and control groups, respectively, did not think that they always have to wash hands after visiting the toilet and 81 (36.8%) and 79 (35.9%) women in the intervention and control groups, respectively, believed that they only need to wash hands with soap when their hands are heavily dirty^10^. Moreover, 94 (42.7%) and 93 (42.3%) in the intervention and control groups, respectively, perceived that washing hands with water alone can remove germs. Another study in a rural setting of northwest Ethiopia also reported that only 65 (17.5%) of women thoroughly rubbed all parts of their hands for at least 20 s, and 44 (11.8%) of the women wiped their hands on their cloth to dry^11^.

Hand hygiene practice at home is affected by many factors. The factors can be categorized in to availability of handwashing infrastructure (such as availability of a handwashing facility and availability of improved latrine), individual motives (such as desire to avoid germs or contamination and desire to care, being taught handwashing behavior from a young age, and family discussion), risk perception (such as perceived that water is effective to remove germs, perceived that washing hands with soap is efficacious in reducing disease transmission, and perceived that hands are clean), social environment (such as having role models in the society), socio-economic factors ( such as education, income, and family size), and healthcare system (such as health education, health supervision, and community health workers)^12–14^. However, there might be other contextual factors that need further investigation. Accordingly, this study was conducted to assess hand hygiene practice and associated factors among rural communities in northwest Ethiopia.

## Methods

### Study design and setting

A community-based cross-sectional study with structured observation was conducted in May 2016 among rural households in Central and North Gondar administrative zones of the Amhara national regional state, Ethiopia. Central Gondar zone covers thirteen districts and North Gondar zone covers seven districts. The total population residing in Central Gondar is estimated to be 2,896,928 and it is estimated to be 912,112 in North Gondar zone^15^. The area is characterized by poor water, sanitation and hygiene conditions were indiscriminate disposal of wastes, limited access to improved drinking water and sanitation services are very common^16^.

### Sample size calculation and sampling procedures

The sample size was calculated using simple population proportion formula with the following assumptions: proportion of rural households who had one or more ectoparasites (p) = 50% since there was no similar study in the area, level of significance (α) = 5%, 95% confidence interval (standard normal probability), z: the standard normal tabulated value, and margin of error (d) = 5%.

$$\mathrm{n} = \frac{{Z\alpha }^{2}p(1-p)}{{d}^{2}}= \frac{{1.96}^{2}*0.5(1-0.5)}{{0.05}^{2}}=384.$$

The final sample size was 1210, with a design effect of 3 and a non-response rate of 5%. All rural households in central and north Gondar administrative zones were considered for sampling. First, we chose four districts and we then selected four kebeles (the lowest administrative unit in Ethiopia) from each district at random using a simple random sampling technique, that is, the lottery method. Finally, we selected 1210 rural households (the analysis unit of this study) using a systematic random sampling technique. We began collecting data in households located on the right side of the local administrators’ office. Assuming that the average number of households in each rural kebele is 200^17,18^, a sampling interval (K = 5) was calculated by dividing 200 by the kebele’s predetermined sample size (n = 43). Following that, a number between one and the sampling interval was chosen at random using the lottery method, which is known as the random start, and was used as the first number included in the sample. Then, after the first random start, every fifth household was sampled until the desired sample size for each kebele was reached.

### Data collection tools and procedures

Data were collected using structured and pretested questionnaire. The questionnaire was prepared based on a review of relevant literature^19–23^. The questionnaire was first prepared in English language and translated to the local Amharic language, and back-translated into English to check consistency. The questionnaire was organized in to three parts: (i) socio-demographic information, (ii) access to hygiene and sanitation information, and (iii) hand hygiene practice. Environmental health experts were participated in the data collection process. Hand hygiene data were gathered by assessing the usual handwashing behavior of households using self-reports. Data collectors also looked at the hands of each family members to see the general cleanliness and conditions of fingernails. To assure the quality of data, the tool was pretested prior to data collection; a one-day training was given to data collectors and field supervisors on the tool, data collection techniques, and ethical issues during data collection; close supervision of data collection; and the filled questionnaires were daily checked for completeness.

### Measurement of outcome variable

Hand hygiene practice in rural communities, the primary outcome variable of the study was considered as “good” if (i) every family member of the households usually wash hands with water and soap or other locally available rubbing agents such as sand, leaves, and ash before preparing foods, before and after eating, after latrine use, and after handling wastes and animals; (ii) every family member dried hands in the air after washing; and (iii) every family member of the households usually kept fingernails clean and short using personal nail cutters^20,21,23^.

### Statistical analyses

Data were entered using EPI-INFO version 3.5.3 statistical package [Centers for Disease Control and Prevention (CDC) in Atlanta, Georgia (USA)] and exported into Statistical Package for Social Sciences (SPSS) version 20 (Armonk, NY: IBM Corp.) for further analysis. For most variables, data were presented by frequencies and percentages. We included predictors to the multivariable binary logistic regression model from the literature regardless of their bivariate p-value to identify factors associated with hand hygiene practice in rural communities. Statistically significant associations were declared on the basis of adjusted odds ratio (AOR) with 95% confidence interval (CI) and p-values < 0.05. Multicollinearity was tested using variance inflation factor (VIF) and model fitness was check using Hosmer and Lemeshow goodness-of-fit test.

### Ethics approval and consent to participate

Ethical clearance was obtained from the Institutional Review Board of the University of Gondar (reference number: V/P/RCS/05/1520/2016). There were no risks due to participation and the collected data were used only for this research purpose with complete confidentiality. Written informed consent was obtained from household heads. All the methods were carried out in accordance with relevant guidelines and regulations.

## Results

### Socio-demographic characteristics

A total of 1190 households participated in the current study, with a response rate of 98.3%. A total of 6089 individuals (3187 males and 2902 females) were surveyed in 1190 households and 513 (43.1%) of the households had more than five family members. Over three quarter, 888 (75.3%) of the female heads did not receive formal education and 643 (59.3%) of the male heads did not attend formal education. The vast majority, 1123 (95.2%) of the female heads were farmers by their occupation and 1045 (96.3%) of the male heads were also farmers (Table 1).

**Table 1:** Sociodemographic characteristics of households (n = 1190) in a rural setting of northwest Ethiopia, May 2016.

Sociodemographic characteristics	Frequency	Percent
Number of individuals surveyed in 1190 households (n = 6089)		
Male	3187	52.3
Female	2902	47.7
Family size of households		
< 5	677	56.9
> 5	513	43.1
Maternal education (n = 1180)		
No formal education	888	75.3
Attend formal education	292	24.7
Paternal education (n = 1085)		
No formal education	643	59.3
Attend formal education	442	40.7
Maternal occupation (n = 1180)		
Farmer	1123	95.2
Merchant	35	3.0
Government employee	8	0.7
Student	3	0.3
Daily laborer	11	0.9
Paternal occupation		
Farmer	1045	96.3
Merchant	22	2.0
Government employee	9	0.8
Daily laborer	9	0.8

### WASH conditions

Five hundred and sixty-five (47.5%) of the households reported that they received hygiene and sanitation education and 967 (81.3%) of the households reported that health professionals closely supervised them. Furthermore, 812 (68.2%) of the households reported that they regularly discussed about health and sanitation issues with their family. The vast majority, 1100 (92.4%) of the households had no access to improved sanitation facilities. About one-fifth, 233 (19.6%) of the households collected drinking water from unimproved sources. Majority, 1154 (97%) of the households had no access to basic water service-level (Table 2).

**Table 2:** Water, sanitation, and hygiene conditions of households (n = 1190) in a rural setting of northwest Ethiopia, May 2016.

WASH conditions	Frequency	Percent
WASH education		
Yes	565	47.5
No	625	52.5
Health professionals’ regular supervision		
Yes	967	81.3
No	223	18.7
The family discussed about hygiene and sanitation		
Yes	812	68.2
No	378	31.8
Access to improved latrine		
No	1100	92.4
Yes	90	7.6
Drinking water sources		
Unimproved	233	19.6
Improved	957	80.4
Volume of water collected		
$\boldsymbol{ }<$ 20 l/c/d	1154	97.0
$\boldsymbol{ }\ge$ 20 l/c/d	36	3.0

### Hand hygiene practices

**Figure 2:**
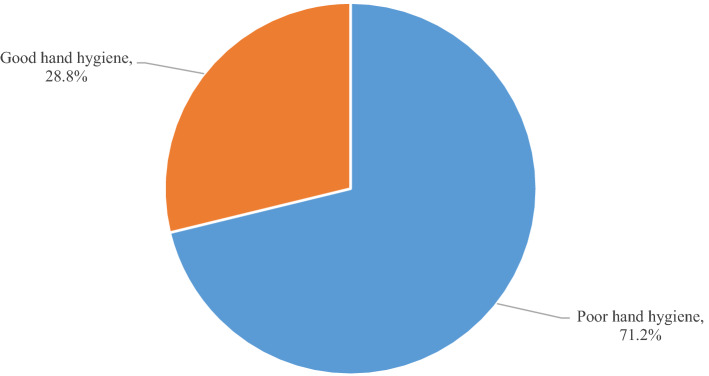
Proportion of households who had good and poor hand hygiene practice in a rural northwest Ethiopia, May 2016.

In the current study, majority of the households reported that all the family members usually washed hands before preparing foods (87.2%), before and after having foods (98.7%), after defecation (77.6%), and after any polluting activities (79.6%). However, about half (47.0%) of the households reported that the family members washed hands with water alone. The most common reported reasons for not using soap or other locally available handwashing agents were expensiveness of soap (21.6%) and negligence (23.6%). Moreover, 29.4% of the households reported that all the family members kept fingernails clean and short using personal nail cutters (Table 3). Overall, 28.8% (95% CI 26.2, 31.4%) of the households had good hand hygiene practice (Fig. 2).

**Table 3:** Hand hygiene practice of households (n = 1190) in a rural northwest Ethiopia, May 2016.

Handwashing practice	Frequency	Percent
Critical times all the family member usually wash hands		
Before and after having foods	1175	98.7
After defecation	923	77.6
Before preparing foods	1038	87.2
After any polluting activities	947	79.6
What households usually used to wash hands		
Water alone	559	47.0
Water and soap	587	49.3
Water and local rubbing agents	44	3.7
Reasons for not using soap or other handwashing agents		
Soap is expensive	257	21.6
Water alone can clean hands	74	6.2
Negligence	281	23.6
All the family members kept fingernails clean and short using personal nail cutter		
Yes	350	29.4
No	840	70.6

### Factors associated with hand hygiene practice

Family size, maternal education, paternal education, access to water, family discussion, health supervision, and health education were the variables entered into the multivariable binary logistic regression model. In the adjusted analysis, paternal education, access to water, family discussion, and health education were significantly associated with hand hygiene practice of households in a rural northwest Ethiopia. The odds of good hand hygiene practice was 1.79 times higher among households headed by male heads who attended formal education compared with their counterparts (AOR: 1.79, 95% CI 1.33, 2.40). Similarly, households who regularly discussed about hygiene and sanitation with their families had 1.56 times higher odds of good hand hygiene practice (AOR: 1.56, 95% CI 1.08, 2.26). The odds of having good hand hygiene practice was 2.23 times higher among households who received health education in three months prior to the survey compared with households who did not receive health education (AOR: 2.23, 95% CI 1.62, 3.06). Households who had access to basic water service level had 3.51 times higher odds of good hand hygiene practice (AOR: 3.51, 95% CI 1.02, 12.05) (Table 4).

**Table 4:** Factors associated with hand hygiene practice of households (n = 1190) in a rural northwest Ethiopia, May 2016.

Variables	Hand hygiene practice		COR with 95% CI	AOR with 95% CI
	Good	Poor		
Family size				
$\le$ 5	192	485	0.95 (0.74, 1.22)	1.00 (0.76, 1.32)
$>$ 5	151	362	1.0	1.0
Maternal education				
No formal education	246	642	1.0	1.0
Attend formal education	94	198	1.24 (0.93, 1.65)	0.93 (0.66, 1.29)
Paternal education				
No formal education	154	489	1.0	1.0
Attend formal education	161	281	1.82 (1.40, 2.37)	1.79 (1.33, 2.40)***
Family discussion				
Yes	271	541	2.13 (1.59, 2.86)	1.56 (1.08, 2.26)*
No	72	306	1.0	1.0
Health supervision				
Yes	291	676	1.42 (1.01, 1.99)	0.70 (0.46, 1.06)
No	52	171	1.0	1.0
Health education				
Yes	218	347	2.51 (1.94, 3.26)	2.23 (1.62, 3.06)***
No	125	500	1.0	1.0
Access to water				
No basic access	337	817	1.0	1.0
Basic access	6	30	2.06 ( 0.85, 5.00)	3.51 (1.02, 12.05)*

Hosmer and Lemeshow test = 0.75, VIF = between 1.009 and 1.198.

*AOR* adjusted odds ratio, *CI* confidence interval, *COR* crude odds ratio.

*Statistically significant at p < 0.05.

***Statistically significant at p < 0.001.

## Discussion

This community-based cross-sectional study was conducted to assess hand hygiene practice and associated factors among rural communities in northwest Ethiopia and found that 87.2% of the households usually washed hands before preparing foods, 98.7% before and after eating, 79.6% after any polluting activities, and 77.6% after defecation. Forty-seven percent of the households washed hands with water alone and 49.3% used water and soap. In the current study, the proportion of households who washed hands before and after having foods, before preparing foods, and after any polluting activities is higher than the proportion of a rural community who washed hands before and after having foods (78%), before preparing foods (93%), and after any polluting activities (55%) in rural Tigray region of Ethiopia^24^ and the proportion of a rural community who washed hands before and after having foods (92.6%), before preparing foods (60.9%), and after any polluting activities (57%) in Davangere Taluk of India^25^. The proportion of households who washed hands with water and soap in the current study is lower than the proportion of a rural community who used water and soap in Davangere Taluk of India (62.5%)^25^ and Pune of India (79.49%)^20^. On the other hand, the proportion of households who washed hands with water alone in the current study is greater than the proportion of a rural community who used water alone in Davangere Taluk of India (28.1%)^25^ and Pune of India (12.53%)^20^. Moreover, in the current study, the proportion of households who washed hands after defecation is lower than the proportion of a rural community who washed hands after defecation in Davangere Taluk of India (100%)^25^ and Pune of India (100%)^20^. In addition, 7.98% of a rural community in Pune of India^20^ and 9.4% of a rural community in Davangere Taluk of India^25^ used water and antiseptic solution. In the contrary, all of the households in the current study did not use antiseptic solution. This low hand hygiene practice in the current study might be explained by many factors such as lack of knowledge, incorrect behavior patterns, insufficient training, heavy workloads, and poverty. In the study area, perception of cleanliness is the major barrier to follow recommended practice of hand hygiene. In the area, households may not use cleaning agents if there is no visible dirt or unpleasant odor. Lack of knowledge about critical times and right technique of hand washing might also explain low hand hygiene practice in the area^10,11^. Moreover, since the rural households are engaged in outdoor activities with heavy workload, they will not have access to handwashing facilities and may not have time in following the steps of effective handwashing as to them it seemed time consuming. In addition, soap is not always available in the area due to cost^10^.

Hand hygiene practice among rural households in northwest Ethiopia was significantly associated with paternal education and provision of health education in the area, which is in agreement with findings of other studies^26–28^. The association between education and hand hygiene practice can be justified that educated household heads may have awareness about the benefits of good hand hygiene practice. Education encourages changes in healthy behaviors and is an effective strategy to promote hygiene and sanitation^29–32^.

The current study depicted that households who regularly discussed about hygiene and sanitation with their families had higher odds of good hand hygiene practice. Family discussion is an effective strategy to promote and sustain hygiene and sanitation practices at individual family member or household level. Family discussion or conversation on hygiene and health is part of health education strategies is associated with increased demand and practice hygiene and sanitation services. For individual family members, habits of personal hygiene are mostly acquired during childhood, and are, therefore, influenced by one’s family. As documented in literature, the family is a key setting for health promotion. Contemporary health promoting family models can establish scaffolds for shaping health behaviors and can be useful tools for education and health promotion^33–35^.

This study also revealed that hand hygiene practice among rural households in area was statistically associated with access to water. Households who had access to basic water service level had higher odds of good hand hygiene practice. This finding is in line with findings of other studies^36–38^. Households with access to water can frequently wash hands whenever contaminated unless the behavioral factors affecting the handwashing practice. In the contrary, water scarcity limits practicing basic hygiene at home. Hand hygiene might not be a priority concern when water is scarce. Families may prioritize using available water for drinking and cooking rather than using it for handwashing in scarcity situations^39–41^.

As limitations, the self-reported data may not be reliable since the study subjects may make the more socially acceptable answers rather than being truthful and they may not be able to assess themselves accurately, which might result reporting bias. Moreover, variables included in the current study to identify factors associated with hand hygiene practices are not complete. As a strength, the study households were selected at random using systematic random sampling technique and so that all rural households in the area had an equal chance to be included in the study and findings of this study will be generalizable. Findings of the study can, therefore, complement the national and local hygiene promotion campaigns. However, the generalizability of results might be affected the aforementioned bias.

## Conclusion

About one-third of the rural households had good hand hygiene practice and more than two-third of the households had poor hand hygiene practice in the study area. Washing hands with soap in different critical times is not commonly practiced in the area and about half of the households used water alone to wash hands. This may imply that hands in the area may play roles in spreading infections in the community. The study found that hand hygiene practice in the area is more of affected by access to health and hygiene information, which may suggest the importance of hand hygiene campaign and health education in the area. People need to be informed about washing hands using cleaning agent and maintaining recommended process at all critical times. They also need to know water alone is not enough to remove germ. Helping people visualize the advantages of good hand hygiene and the dangers of poor hand hygiene using visual aids like flash card or video could be helpful. Also, the use of local rubbing agents such as sand, ash, and leaves can be promoted in the area in the absence of soap. Moreover, behavioral factors that hider effective handwashing practices in the area need to be investigated.

## Data Availability

The datasets generated during and/or analyzed during the current study are available from the corresponding author on reasonable request**.**
